# Relationship between acceleration of axial elongation and ocular biometry in schoolchildren

**DOI:** 10.1007/s10384-025-01227-x

**Published:** 2025-06-17

**Authors:** Takehiro Yamashita, Hiroto Terasaki, Takuto Hamada, Ryo Asaoka, Naoya Yoshihara, Naoko Kakiuchi, Taiji Sakamoto

**Affiliations:** 1 Department of Ophthalmology, Kagoshima University Graduate School of Medical and Dental Sciences, Kagoshima, Japan; 2 Department of Ophthalmology, Seirei Hamamatsu General Hospital, Shizuoka, Japan; 3 Seirei Christopher University, Shizuoka, Japan; 4 Nanovision Research Division, Research Institute of Electronics, Shizuoka University, Shizuoka, Japan; 5 The Graduate School for the Creation of New Photonics Industries, Shizuoka, Japan

**Keywords:** Axial length, School myopia, Ocular enlargement, Anterior chamber depth, Axial elongation

## Abstract

**Purpose:**

The speed of axial elongation typically slows during the growth phase; however, in some eyes, it accelerates, leading to myopia progression during school age. This study examined the association between ocular biometrics and the acceleration of axial elongation (AAE) in children.

**Study design:**

Longitudinal, prospective, observational study

**Methods:**

This cohort study included 67 right eyes of elementary school children, tracked over six years (from ages 8.5 to 14.5). Annual measurements were conducted for optical axial length, anterior chamber depth, and lens thickness. Yearly axial elongation was calculated for each time period, and AAE was estimated using regression analysis coefficients. Spearman's correlation was used to evaluate the association between AAE and ocular biometric parameters measured in the first year.

**Results:**

The average axial length in the initial year was 23.37 ± 0.89 mm. By the sixth year, the mean axial elongation reached 1.50 ± 0.49 mm, while the average AAE was recorded as -0.015 ± 0.048. AAE was significantly correlated with first-year axial length (r = − 0.40, *p* < 0.001), anterior chamber depth (r = 0.33, p = 0.007), and lens thickness (r = − 0.42, *p* < 0.001).

**Conclusion:**

Some eyes with hyperopic ocular biometry at 8.5 years of age exhibited accelerated axial elongation during school age. Future research on myopia should consider both acceleration and axial elongation.

## Introduction

Myopia develops as the eyeball grows. Eyeball enlargement has been observed in both fetuses and young adults. The axial length (AL) of the eye, approximately 5 mm at 12 weeks of gestational age, expands rapidly in utero [1–3], reaching 17.6 ± 0.87 mm in newborns at an average gestational age of 38.4 weeks [4]. By the age of 3 years, the AL increases to 21.73 ± 0.69 mm [4], after which its growth gradually slows and typically halts by the age of 20 years [5]. Longer AL at birth is associated with greater axial elongation during the first three years of life [4], suggesting common factors influencing axial elongation from the fetal stage to early childhood.

In infancy, the primary factors affecting axial elongation are intraocular pressure (IOP) and eye-wall elasticity [6, 7]. After the age of 3 years, IOP gradually decreases, the ocular wall becomes stiffer, and axial elongation slows [8, 9]. During school age, the growth of the anterior eye gradually ceases, while the posterior eye elongates continuously, resulting in progressive myopia [10, 11]. Myopia progresses relatively rapidly until around the age of 10 years in eyes with an originally long AL, but the rate of axial elongation per year slows thereafter [8–10]. However, studies investigating axial elongation in children in Germany and Taiwan have found that myopia progresses rapidly from around the age of 10 years [12, 13]. This suggests that some eyes with slower axial elongation before the age of 10 years may experience accelerated axial elongation due to other factors. Numerous studies identify predictors of the onset and progression of myopia [14]. Ocular biometric parameters, including refraction, axial length (AL), anterior chamber depth (ACD), lens thickness (LT), vitreous chamber depth, and corneal curvature radius from the first year, have been identified as predictive factors [15, 16]. Lifestyle factors, including near work, outdoor activities, and education, have also been identified [17, 18]. Additionally, other factors such as age, ethnicity, sex, height, visual acuity, parental refraction, and education have been shown to influence myopia progression [19, 20]. These studies have primarily examined factors affecting changes in refraction and AL. If annual eye growth gradually slows and eventually ceases, investigating the total amount of change in AL and refraction would suffice. However, if new factors emerge during school age that accelerate axial elongation, it becomes necessary to investigate the acceleration and deceleration of axial elongation to identify the distinct factors influencing axial elongation during this critical period.

In our 2-year cohort study of children, we found that at the age of 8.5 years, eyes with hyperopic tendencies characterized by a shallower ACD, thicker LT, and shorter AL exhibited accelerated axial elongation per year [21]. Consequently, the axial elongation in the second year was greater than in the first year. However, this study covered only a 2-year period, while previous reports indicate that this acceleration of axial elongation persists beyond this timeframe [12]. This highlights the need for confirmation through longer-term studies. To address this gap, we extended the follow-up period of the previously reported 2-year case series by an additional 4 years to determine whether similar results could be observed over the longer term. This study aimed to quantify the acceleration of axial elongation (AAE) using regression analysis of axial elongation per year and to investigate its correlation with ocular biometrics measured at age 8.5 years.

## Material and methods

### Ethics statement

This study adhered to the principles outlined in the Declaration of Helsinki and received approval from the Ethics Committee at Kagoshima University Hospital (Approval ID: 170116 (643)). Written informed consent was obtained from all participants and their guardians. Additionally, this research was registered in the University Hospital Medical Network Clinical Trials Registry (Registry ID: UMIN000015239).

### Participants

This prospective, longitudinal observational study included third-grade students, aged 8 to 9 years, from the Elementary School of the Faculty of Education at Kagoshima University during the initial assessment [21]. Of 144 third-year students, informed consent was obtained in the first year from the parents of 122 (87.4%) students. Participants underwent examinations from November 17 to December 18, 2014, during the first year and were subsequently assessed during the same period each year for 7 years. Color fundus images were captured using the 3D optical coherence tomography (OCT)-1 Maestro (Topcon), while AL, ACD, and LT were measured using an OA-2000 Optical Biometer (Tomey). Eyes with known ocular diseases were excluded based on medical history, fundus photography, and OCT findings. No eyes had a history of ocular disease or developed ocular disease during the observation period. Twenty-two eyes were excluded due to difficulties in measuring ACD and LT [22]. Additionally, 33 students were excluded from the study due to absenteeism, transfer to another school, or enrollment in a different junior high school. Finally, the right eyes of 67 students were analyzed.

### Calculation of total AL elongation and acceleration of axial elongation

**Fig. 1:**
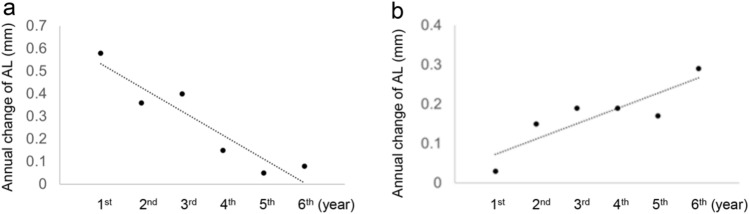
Quantifying total axial length elongation (TALE) and acceleration of axial elongation (AAE). TALE was calculated by subtracting the first-year axial length (AL) from the last year's AL. The annual change in AL was calculated for each year. AAE was quantified using the regression analysis coefficient. A negative AAE value indicates deceleration (a; − 0.11 mm/year), and a positive AAE value indicates acceleration (b; +0.04 mm/year) in the AL elongation rate

AL, ACD, and LT were measured annually over 7 years without the use of cycloplegia. The total axial length elongation (TALE) was calculated by subtracting the AL measured in the first year from that measured in the final year. The yearly axial elongation, represented by black arrows, was calculated for each individual year. The AAE was quantified using the coefficient of regression analysis. A negative AAE value indicated deceleration (Fig. 1a), while a positive AAE value indicated acceleration (Fig. 1b) of the AL elongation rate.

### Statistical analyses

Statistical analyses were performed using SPSS Statistics 21 for Windows (IBM Corp.). The coefficient of regression analysis was employed to quantify the AAE. Spearman's rank correlation was applied to assess the relationships between the ocular biometric measurements (AL, ACD, and LT) taken in the first year and the TALE and AAE values.

## Results

The participants included 33 boys and 34 girls. The mean ± SD and range of the first-year ACD were 3.62 ± 0.23 mm (2.96 to 4.03 mm), the first-year LT was 3.52 ± 0.16 mm (3.18 to 3.88 mm), the first-year AL was 23.39 ± 0.92 mm (20.52–25.80 mm), and the seventh-year AL was 24.91 ± 1.12 mm (21.87–27.12 mm). The mean ± SD and range of TALE were 1.52 ± 0.50 mm (0.35 to 2.44 mm), and the AAE was − 0.02 ± 0.05 mm/year (-0.15 to 0.08 mm/year) (Table 1). Forty-one eyes exhibited decelerated axial elongation, while 26 eyes showed accelerated axial elongation from 8.5 to 14.5 years of age.

**Table 1.** Ocular biometrics of participants

Ocular biometrics	Mean ± standard deviation (range)
1^st^ year ACD (mm)	3.62 ± 0.23 (2.96–4.03)
1^st^ year LT (mm)	3.52 ± 0.16 (3.18–3.88)
1^st^ year AL (mm)	23.39 ± 0.92 (20.52–25.80)
7^th^ year AL (mm)	24.91 ± 1.12 (21.87–27.12)
TALE (mm)	1.52 ± 0.50 (0.35–2.44)
AAE (mm/year)	-0.02 ± 0.05 (-0.15 to 0.08)

*AL* axial length; *ACD* anterior chamber depth; *AAE* acceleration of axial elongation; *LT* lens thickness; *OCT* optical coherence tomography; *TALE* total axial length elongation

**Fig. 2:**
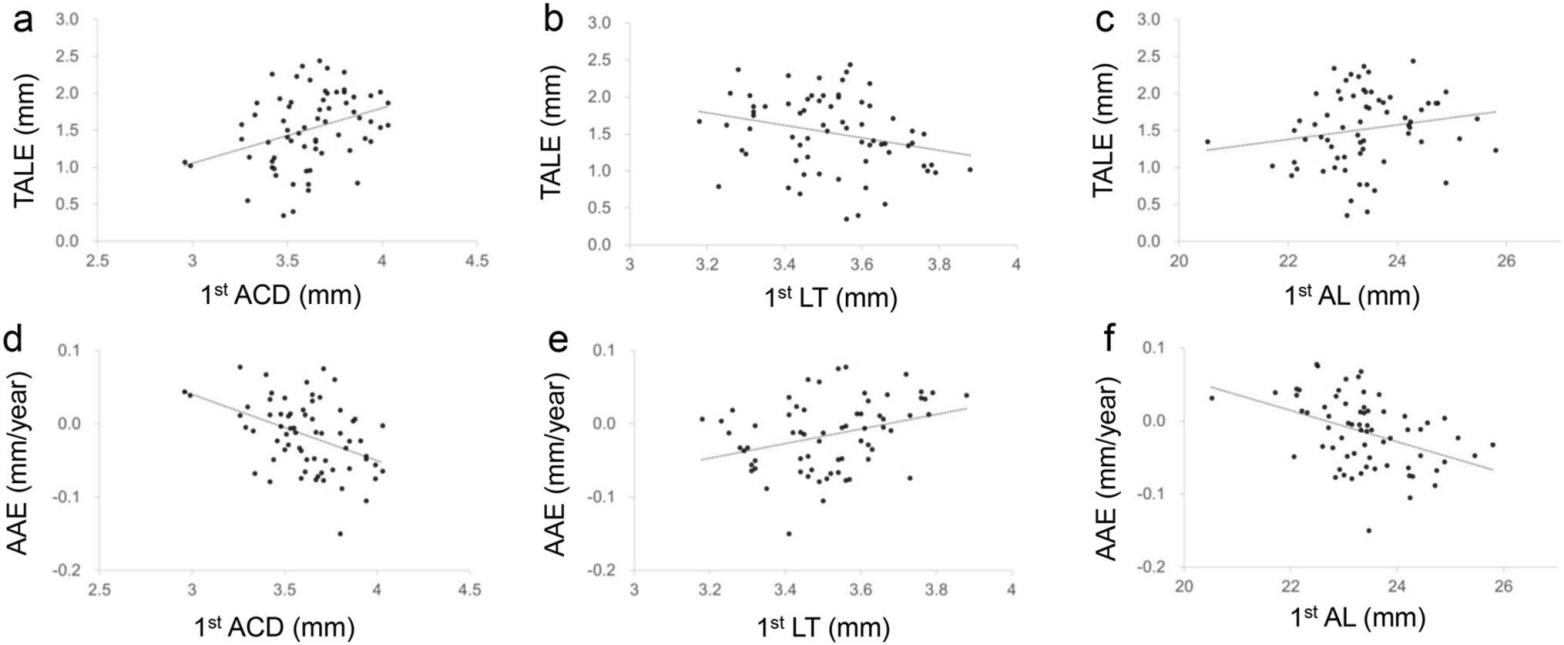
Scatter plot of first-year ocular biometrics (anterior chamber depth (ACD), lens thickness (LT), axial length (AL)) and total axial length elongation (TALE) (a, b, c), or acceleration of axial elongation (AAE) (d, e, f)TALE was significantly correlated with first-year ACD (a; r = 0.36, *p* = 0.002) and LT (b; r = − 0.27, *p* = 0.020) but not with AL (c; r = 0.22, p = 0.069). AAE was significantly correlated with first-year ACD (d; r = − 0.42, *p* < 0.001), LT (e; r = 0.33, *p* = 0.007), and AL (f: r = − 0.44, *p* < 0.001)

TALE was significantly correlated with the first-year ACD (r = 0.36, p = 0.002) and LT (r = − 0.27, p = 0.029) but not with AL (r = 0.22, p = 0.069) (Fig. 2a, b, c). AAE was significantly correlated with the first-year ACD (r = − 0.42, p < 0.001), LT (r = 0.33, p = 0.007), and AL (r = − 0.44, p < 0.001) (Fig. 2d, e, f).

## Discussion

The results demonstrate that eyes with a shallower ACD, thicker LT, and shorter AL at the age of 8.5 years tended to exhibit greater acceleration of AAE. A similar trend was observed in our previous 2-year cohort study [19], suggesting that hyperopic ocular biometry at the age of 8.5 years is associated with accelerated axial elongation during school age. This finding implies that myopia progression may be more pronounced in eyes with greater AAE during this period. Growth curves of AL from previous studies [12, 13] also reveal a slight upward trend around the age of 10 years, consistent with the presence of some eyes in which axial elongation accelerates.

Conversely, myopic ocular biometry at the age of 8.5 years was associated with decelerated axial elongation during this period. In our 2-year cohort study, eyes with deeper ACD, thinner LT, and longer AL at the age of 8.5 years had significantly greater TALE [21]. In this 6-year cohort study, a comparable correlation was found among ACD, LT, and TALE. However, the association between AL at 8.5 years of age and TALE did not attain statistical significance. These findings suggest that the rate of annual axial elongation observed up to the age of 8.5 years undergoes a gradual reversal during school age. A novel finding of this study is that eyes with hyperopic ocular structure at the age of 8.5 years exhibit an accelerated rate of axial elongation, as demonstrated by regression analysis, during school age. This underscores the importance of early identification of hyperopic ocular biometry for predicting and managing myopia development in children of school age.

These trends were observed not only during the two years following the age of 8.5 years [19] but also over the subsequent six years, up to the age of 14.5 years. Since axial elongation typically ceases at adulthood, eyes exhibiting accelerated elongation are expected to eventually slow down and stop. Future research is needed to quantify the acceleration of axial elongation from the age of 15 years to adulthood. Notably, this study found very few eyes that deviated significantly from the regression line in Figure 2f. Eyes located in the upper right of the scatter plot represent those with accelerated axial elongation despite already having a long axial length at 8.5 years of age. Conversely, those in the lower left represent eyes with decelerated axial elongation despite a shorter initial axial length at 8.5 years of age. Such deviations may indicate the development of special eye shapes, and this scatter plot could be valuable in predicting eye shape-related diseases, such as pathological myopia and angle-closure glaucoma.

Explaining this difference is challenging with the current data, but some insight may be drawn from past literature and other sources. Factors such as shorter AL, shallower ACD, and thicker LT are associated with an increased risk of primary angle closure [23]. In primary angle closure, posterior chamber pressure exceeds anterior chamber pressure, causing anterior bowing of the iris. Conversely, deeper ACD, longer AL, and thinner LT are risk factors for pigment dispersion syndrome, where higher anterior chamber pressure leads to posterior iris bowing [23]. Iris curvature varies in vivo due to differences in pressure between the anterior and posterior chambers [24]. Shah et al. investigated iris curvature in boys aged 10 to 12 years utilizing anterior segment OCT. They found posteriorly curved (concave) irises in some eyes and anteriorly curved (convex) irises in others [25]. These variations occur around age 10 when the lens and ocular tissues begin to harden [26]. Variations in pressure between the anterior and posterior chambers could potentially impact axial elongation rates during this developmental stage.

Although no studies have directly examined the relationship between iris curvature and axial elongation, numerous papers have explored the association between IOP and the onset and progression of myopia. While longitudinal studies on the relationship between AL and IOP in early childhood are lacking, evidence from research on childhood glaucoma suggests that AL is highly sensitive to changes in IOP [27, 28]. The Collaborative Longitudinal Evaluation of Ethnicity and Refractive Error (CLEERE) study reports that mean IOP prior to the onset of myopia onset was markedly higher in children compared to emmetropic peers, suggesting that IOP may influence the onset of myopia [29]. In contrast, the Correction of Myopia Evaluation Trial (COMET), a 5-year follow-up study beginning at the age 6 years, found no significant association between IOP and myopia progression or changes in AL [30]. Conversely, the Anyang Childhood Eye Study indicates that IOP, as measured by noncontact tonometry, was approximately 1 to 2 mm Hg higher in myopic eyes than in emmetropic or hyperopia eyes. However, in eyes with greater myopia progression, IOP was considerably lower, suggesting that progressing myopic eyes may have higher scleral compliance [31]. While elevated IOP appears to contribute to axial elongation during infancy, this effect diminishes in school-age children, and the association may even reverse. This reversal can be attributed to two factors. First, IOP is typically measured through the cornea, and in adults, thinner corneas often result in underestimation of the true IOP [32]. Although children’s corneas are generally thicker and softer than of adults, a similar phenomenon is believed to exist [28]. Therefore, in myopic eyes—where the cornea tends to be thinner—the measured IOP is lower than the true IOP. This weakens the positive association between IOP and axial elongation. Another possible explanation is the reversal phenomenon observed in this study, where axial elongation accelerates in eyes with hyperopic ocular biometry and decelerates in eyes with myopic ocular biometry in school-age children. According to the pupillary block hypothesis, anterior chamber and vitreous pressures are equal prior to the onset of pupillary block; thus, elevated IOP may contribute to myopia development in infancy. However, when pupillary block occurs in school-age children, the measured IOP primarily reflects anterior chamber pressure, while vitreous pressure undergoes a reversal. Specifically, in eyes with hyperopic ocular biometry, when pupillary block occurs, the vitreous pressure becomes higher than the measured IOP, leading to posterior segment elongation even if the IOP reading is low. The opposite phenomenon occurs in eyes with myopic ocular biometry. Therefore, the pupillary block hypothesis may help explain the previously reported reversal phenomenon: high IOP promotes axial elongation in infancy, but lower IOP is associated with myopia progression as children get older. Future long-term cohort studies are needed to investigate this further, ideally incorporating measurements of corneal thickness—which influences IOP readings— and iris curvature, which may serve as an indirect indicator of pressure differences between the anterior and posterior chambers.

This study had several limitations. First, we examined AL rather than refractive errors. Cycloplegia is required for stable measurements of refraction, ACD, and LT. However, the ethics committee did not approve the use of cycloplegics, and the limited examination time necessitated focusing on AL without measuring refractive error. Despite this limitation, axial elongation is a significant contributor to school myopia progression in East Asian populations [33–35], and AL measurement is a reliable method for monitoring myopia progression in schoolchildren [36–39]. Additionally, predicting axial elongation using ACD, LT, and AL without cycloplegia has practical applications in clinical settings. Second, as a long-term observational study, the number of participants decreased from 122 in the first year to 67 in the final analysis, which may have influenced the results. Furthermore, this study did not account for factors such as near vision and outdoor activities, which are known to affect axial elongation in school-aged children. Third, the explanation forwarded here is theoretical, with no supporting data to substantiate it. However, throughout the history of science, many phenomena have been reported and explained theoretically, only to be later substantiated and initiate advancements. Thus, this explanation may still hold significance. Currently, no method exists to directly measure posterior chamber, vitreous, or anterior chamber pressure. The time required to flatten the cornea in a non-contact tonometer and the force required to flatten the cornea in a contact tonometer are converted into IOP by correlation. Estimating pupil block and reverse pupil block forces may be possible using ocular biometrics, but further research is necessary. One drawback is that that these results were derived from Japanese children, a population with one of the highest myopia prevalence rates worldwide [40]. Therefore, the findings may not be generalizable to other populations.

In conclusion, the rate of axial elongation tended to accelerate in eyes with hyperopic ocular biometrics at the age of 8 years. Future myopia research should focus on both the acceleration and total elongation of AL.

## Data Availability

Data are available upon reasonable request.
